# Energy storages on the ferroelectric microstructures with transformation and nano vortex pattern

**DOI:** 10.1038/s41598-024-81318-w

**Published:** 2025-03-03

**Authors:** Han Zheng, Dongxu Pan, Zhengfa Li

**Affiliations:** Physics Department, Shihezi University, Shihezi, 8320003 China

**Keywords:** Energy science and technology, Materials science, Physics

## Abstract

The energy storage and conversion in ferroelectrics can be realized through the microstructures of polar domains and domain walls, which resulting in the transformations from macro/microdomains to nanodomains or forming complex polar topologies. The physical basic models are adopted with domains and domain walls including 90^o^, 180^o^, 71^o^ and 109^o^ which are classified into two categories of 180^o^ and *α*-angle, and are reconstructed with equivalent circuits simplified according to the reported patterns. Although electrical energy is known to be maintained by the charging capacitor, the energy storage effect on ferroelectric microstructure has been rarely explored for the relative paucity of experimental patterns reported with domains and domain walls. The diagrammatic sketches of transformation into nanodomain and vortex pattern are designed, and their respective formulas of total capacitances and energy densities are derived with crucial structural features. The findings reveal novel mechanisms of the relationship between energy storage and microstructures, that may be used to propose effective creation strategies or to design modern measure equipment in future.

## Introduction

With the rapid advancement of science and technology, and more attention on environmental protection, energy storage has become a hot research field. Scientists and engineers have been working together to develop environment-friendly high-efficiency energy storage materials including relaxor ferroelectrics and anti-ferroelectrics and experimental technology^1–6^. Especially because of the superior capacity of energy storage, ferroelectric capacitors have been gotten lots of attention and wide utilization in civil, military, scientific research and other domains. Due to its properties of high energy density *w*_rec_, wide operating temperature range △*T*, quick charge-discharge ability and extended active life *τ*, ferroelectrics is a kind of prospective and promising energy storage material^7–13^. Such as one NaNbO_3_-based relaxor ferroelectric ceramics has been developed by incorporating Bi_2_O_3_^14^, which can not only optimized its polarization characteristics but also significantly enhanced its breakdown strength and energy storage efficiency. And another relaxor anti-ferroelectric ceramic system has been achieved for 0.76NaNbO_3_-0.24Na._5_Bi_0.5_TiO_3_ with a high *w*_rec_ of 12.2 J cm^− 3^ and a moderate *η* of 69% under 680 kV cm^− 1^^15^, which is promising candidate for the next generation of pulse power capacitors.

Up until now, developing ferroelectric energy storage materials with high energy storage density and efficiency even excellent energy storage stability is to meet the demand for growing electricity and integrated applications of renewable energy^16–20^. To deep understand and optimally design the energy storage properties of dielectrics with the ferroelectric nano-to-macro structural transformation and nano vortex pattern, the equivalent circuit models are adopted according to different structural properties of domains and domain walls, and analyzed step by step from statically basic model to complex ones with fixed frequencies, temperatures, and external electric field. From the capacitor with parallel plates, energy storage density (*w*_e_) can be obtained from the following formula with the determined capacitance (*C*) and applied electric field (*E*)

1 $$\:{w}_{\text{e}}=\frac{{W}_{\text{e}}}{\text{V}}=\frac{{\int\:}_{\text{v}}^{}\frac{1}{2}{\upepsilon\:}{\text{E}}^{2}\text{d}\text{V}}{\text{V}}=\frac{{{\upepsilon\:}}_{0}{{\upepsilon\:}}_{\text{r}}{\text{E}}^{2}}{2}=\frac{\text{C}{\text{d}\text{E}}^{2}}{2\text{S}}$$

Here, *W*_e_ is the electric energy stored, *V* is the volume of dielectric in capacitor, and *S* is the opposing area of plates, *ε*_0_ is the permittivity of vacuum, and *ε*_*r*_ is the relative permittivity of ferroelectric material. From the above Eq. (1), the capacitances of ferroelectrics with domain and domain wall are important parameters.

Obviously, for the spatial configuration of domains and domain walls may be has significant relationship with the total capacitance, its effect on energy storage is needed to research. Therefore, the results on circuit analysis can be used to explain and even predict the energy storage performances of capacitors, so as to provide more powerful guidance on the further improvement of designed materials^21^.

## Model and discussion

The microstructure of crystal is related to its crystal system. The angles between polarization directions of different ferroelectric domains not only can be 180^o^ or 90^o^, but also may be 71^o^ or 109^o^. As an example, rhombohedral BiFeO_3_ crystal has the domain walls of 71^o^, 109 ^o^ and 180 ^o^^22^. Therefore, two classified categories of 180 ^o^ and *α*-angle are used, in order to explore the behavior of dielectric domains and domain walls in equivalent circuits and to investigate their effects on energy storage. In the equivalent circuit for *α*-angle domain wall, the domain wall and domain between two pole plates of the equivalent capacitor are not necessarily in contact with both ends, thus they can be divided into three cases to be discussed: (1) both ends out of contact, (2) both ends in contact and (3) one end in contact^23^.

**Fig. 1:**
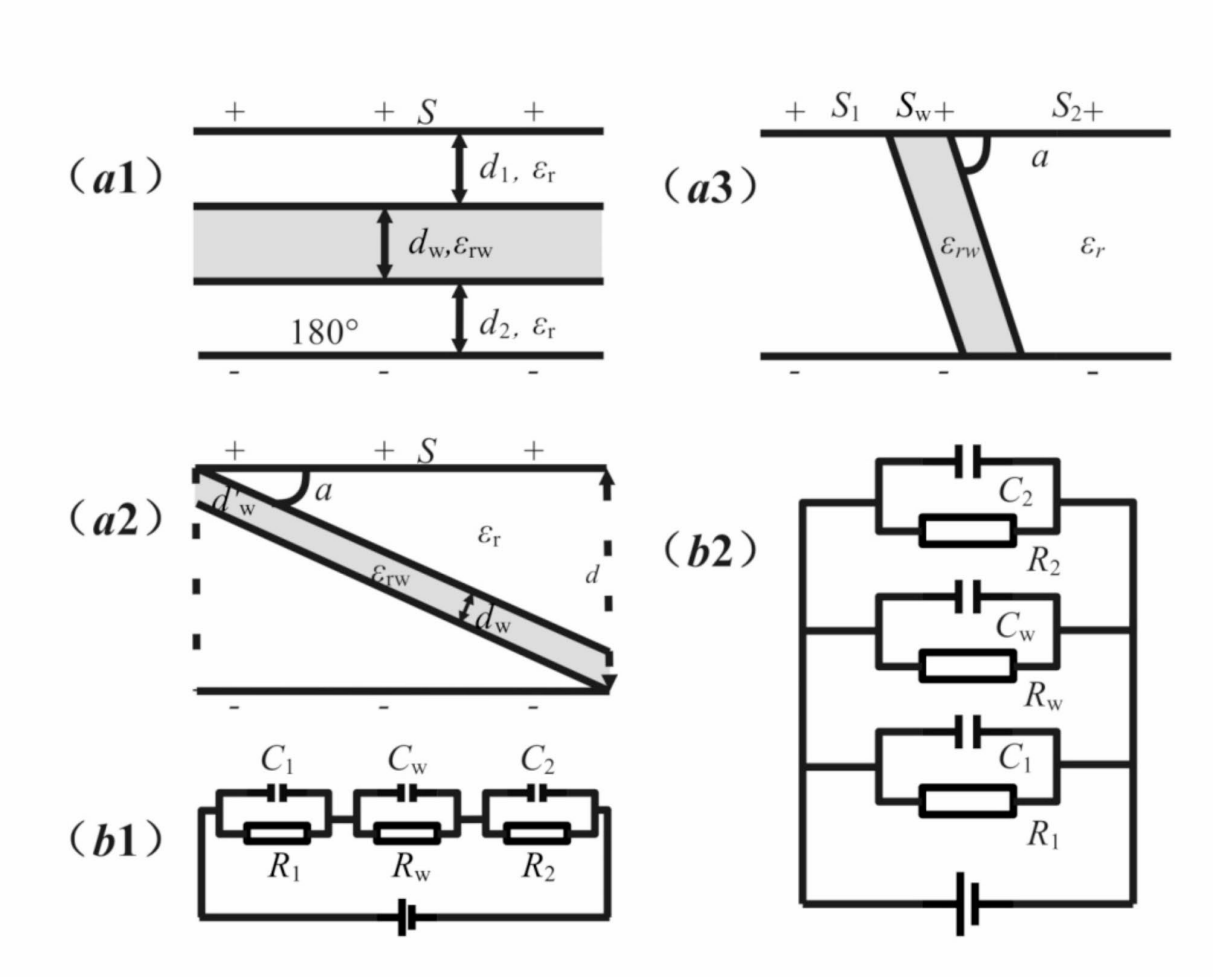
(**a1**,** a2** &** a3**) are thumbnail pictures of 180^o^ and *α*(= 71^o^, 109^o^ or 90^o^) domain and domain wall with both ends in contact and neither ends in contact; (**b1** &** b2**) are the equivalent circuit diagrams of (**a1**,** a2**) and** a3**, respectively.

Figure 1 illustrates the models of 180° and *α*-angle between domains and domain walls with both ends in contact and out of contact, and their equivalent circuits, respectively. Considering the equivalent circuit of *α*-angle between domain and charged domain wall, in which both ends are in contact with pole plates for Fig. 1(a3), when projecting the domain wall in the horizontal direction, it can be neglected since there is only its projection in the direction perpendicular to the poles of electrode plate. Its projection has an equivalent opposite area *S*_*w*_, a distance *d* between the two poles of the capacitor and relative permittivity *ε*_*rw*_. In Fig. 1(a3), the equivalent total capacitor with charged domain walls can be considered containing three small equivalent capacitors connected in parallel, as shown in Fig. 1(b2). For the *α*-angle between domains and charged domain walls which are non-contact at both ends in Fig. 1(a2), if the domain walls are projected along the parallel direction to electrode pole plates, no area with the poles is contacted, and one total equivalent capacitor consisting of three sub-capacitors in series can be viewed, which equivalent circuit diagram is similar to that of 180^o^. Therefore, the equivalent circuits of 180^o^ and *α*-angle between domains and domain walls for both ends out of contact can be viewed as three equivalent sub-capacitors in series containing one of domain wall, as shown in Fig. 1(b1). And the equivalent circuits of *α*-angle for both ends in contact between domains and domain walls can be viewed as three equivalent sub-capacitors in parallel containing one of domain wall as shown in Fig. 1(b2)^23–26^, from which the expressions of their respective equivalent capacitances *C* and energy density *w*_*e*_ can be deduced as follows.

2 $$\:{C}_{\text{F}1\text{a}1}={C}_{180^\circ\:}=\frac{{C}_{1}{C}_{w}{C}_{2}}{{C}_{1}{C}_{2}+{C}_{w}{C}_{2}+{C}_{1}{C}_{W}}=\frac{{\epsilon\:}_{0}S}{\left(\frac{{d}_{1}+{d}_{2}}{{\epsilon\:}_{r}}+\frac{{d}_{w}}{{\epsilon\:}_{rw}}\right)}$$

3 $$\:{w}_{e\text{F}1\text{a}1}={w}_{e180^\circ\:}=\frac{{\epsilon\:}_{0}d{E}^{2}}{2\left(\frac{{d}_{1}+{d}_{2}}{{\epsilon\:}_{r}}+\frac{{d}_{w}}{{\epsilon\:}_{rw}}\right)}$$

4 $$\:{C}_{\text{F}1\text{a}2}=\frac{{C}_{1}{C}_{w}{C}_{2}}{{C}_{1}{C}_{2}+{C}_{w}{C}_{2}+{C}_{1}{C}_{W}}=\frac{{\epsilon\:}_{0}S}{\left(\frac{d-{d}_{w}^{{\prime\:}}}{{\epsilon\:}_{r}}+\frac{{d}_{w}}{{\epsilon\:}_{rw}}\right)}$$

5 $$\:{w}_{e\text{F}1\text{a}2}=\frac{{\epsilon\:}_{0}d{E}^{2}}{2\left(\frac{d-{d}_{w}^{{\prime\:}}}{{\epsilon\:}_{r}}+\frac{{d}_{w}}{{\epsilon\:}_{rw}}\right)}$$

6 $$C_{{F1a3}} = \varepsilon _{0} \frac{{\varepsilon _{r} S_{1} + S_{2} + \varepsilon _{{rw}} S_{w} }}{d}$$

7 $$w_{{eF1a3}} = \frac{{\varepsilon _{0} E^{2} }}{{2S}}\left[ {\varepsilon _{r} S_{1} + S_{2} + \varepsilon _{{rw}} S_{w} } \right]~$$

Where *d*_1_, *d*_2_, *d*_*w*_ are the distances of each equivalent sub-capacitor pole plate in domains or domain walls with *d*_*w*_=*d*^*’*^_*w*_cos*α*, and *S* is the opposite area with *S* = *S*_1_ + *S*_*w*_+*S*_2_. Here *ε*_0_ is the dielectric constant of vacuum, *ε*_*r*_ is the relative one of material, and *E* is the total electric field applied on the pole plates. Additionally, the subscripts of F1*a*1, F1*a*2 and F1*a*3 in capacitance *C* and energy density *w*_*e*_ are noted respectively according to Fig. 1(a1), Fig. 1(a2) and Fig. 1(a3). And the similar subscripts in subsequent formulas are also named by the same way.

**Fig. 2:**
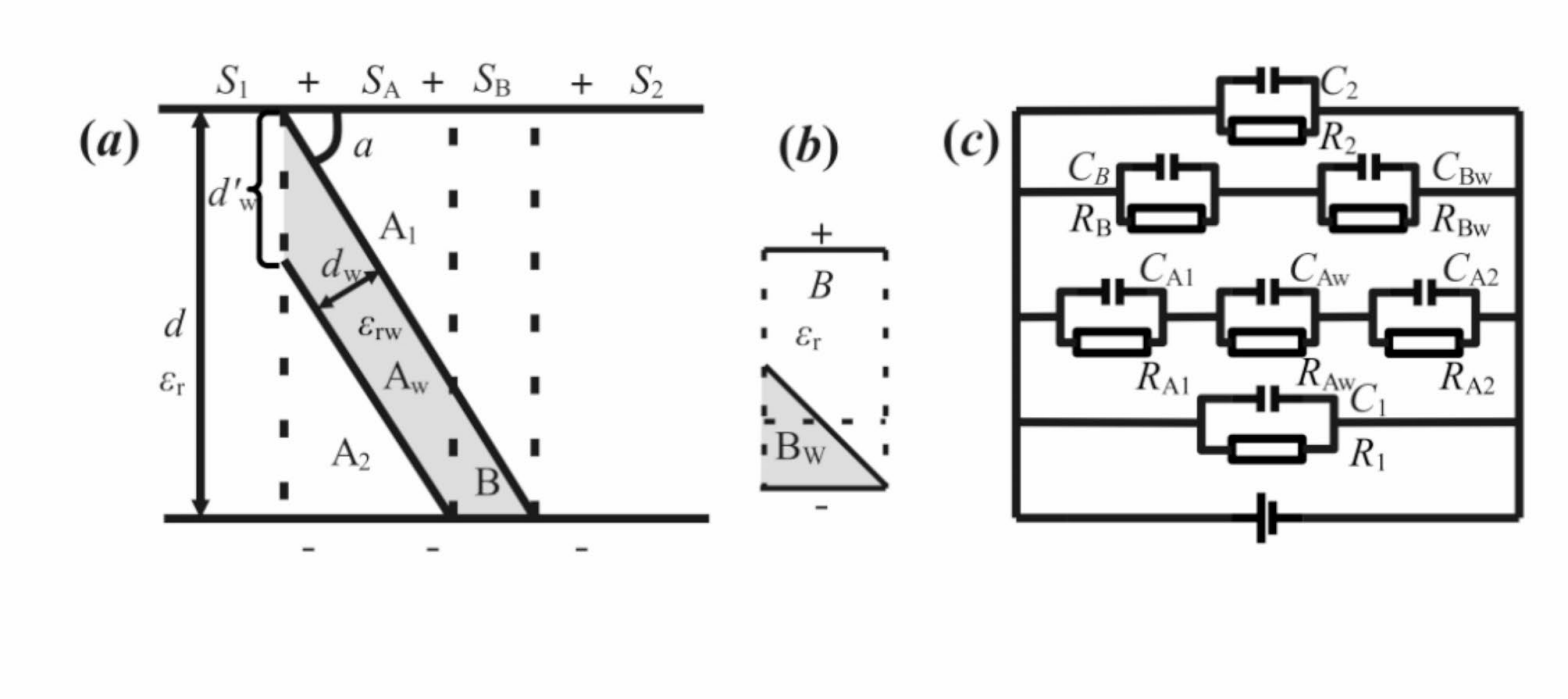
(**a**) is the diagram of regions by division based on the model of *α*(= 71° or 109°) domain and domain wall with one end in contact and the other not, (**b**) is the amplified part of domain wall in region B; and (**c**) is the equivalent circuit diagram of (**a**).

Another configuration is complicated as shown as Fig. 2(a), in which one end contacts pole plate and the other is non contacted with *α*-angle between domain and domain wall, and it maybe belongs to more common situation. According to the relative area of domain wall and domain in Fig. 2(a), the total dimension can be classified into four regions firstly. For distinguishing obviously, the structural difference of domain walls and domain are shown in Fig. 2(a). In which the region B can be regarded as a domain if only considering the domain wall of region A, resulting in one bigger domain connected and formed by region B and region 2. The domain wall can be projected in the direction parallel to the electrode plane, and the results are the same as that of above *α*-angle charged domain wall in Fig. 1(a2). For their ends are not in contact, the corresponding equivalent circuit can be equated with three capacitors connected in series, to instead of the direct use of non-contact *α*-angle charged domain walls both in equivalent circuit and in the formula.

If the size difference between domain wall and domain is small enough in Fig. 2(a), the part B_w_ cannot be ignored in region B. As the same treatment of region 1, A and 2 taken above, in region B the small triangle part B_w_ of domain wall is taken in a similar way, equating to cut off its half height parallel to electrode plane and to supplement it on the right as Fig. 2(b). By the transformation, an equivalent capacitor with a distance *d*^*’*^_*w*_/2 and equivalent positive area *S*_B_ is shown in Fig. 2(b). It is confirmed that the equivalent capacitor of triangle B_w_ is connected in series with that of the left area, and as a whole one is further connected in parallel with those of the other three regions, shown in Fig. 2(c). The total equivalent capacitance *C*_F2a_ and energy density *w*_*e*F2a_ can be expressed as follows.

8 $$\:{C}_{\text{F}2\text{a}}={\epsilon\:}_{0}\left[\frac{{\epsilon\:}_{r}}{d}\left(S-\frac{d}{tan\alpha\:}\right)+\frac{{\epsilon\:}_{r}{\epsilon\:}_{rw}\left(\frac{d}{tan\alpha\:}-{S}_{B}\right)}{{\epsilon\:}_{r}{d}_{w}^{{\prime\:}}+{\epsilon\:}_{rw}\left(d-{d}_{w}^{{\prime\:}}\right)}+\frac{2{\epsilon\:}_{r}{\epsilon\:}_{rw}{S}_{B}}{{\epsilon\:}_{r}{d}_{w}^{{\prime\:}}+{\epsilon\:}_{rw}\left(2d-{d}_{w}^{{\prime\:}}\right)}\right]$$

9 $$w_{{eF2a}} = \frac{{\varepsilon \:_{0} E^{2} d}}{{2~S}}\left[ {\frac{{\varepsilon \:_{r} }}{d}\left( {S - \frac{d}{{\tan \alpha \:}}} \right) + \frac{{\varepsilon \:_{r} \varepsilon \:_{{rw}} \left( {\frac{d}{{\tan \alpha \:}} - S_{B} } \right)}}{{\varepsilon \:_{r} d_{w}^{\prime } + \varepsilon \:_{{rw}} \left( {d - d_{w}^{{\prime \:}} } \right)}} + \frac{{2\varepsilon \:_{r} \varepsilon \:_{{rw}} S_{B} }}{{\varepsilon \:_{r} d_{w}^{{\prime \:}} + \varepsilon \:_{{rw}} \left( {2d - d_{w}^{\prime } } \right)}}} \right]$$

Where, in the physical model containing multiple domain walls, it is assumed that all the dielectric constants of polar domains are set as *ε*_*r*_ and those of domain walls are set as *ε*_*rw*_. The domain walls are all supposed to be in contact with electrode at one end rather than the other end, and the distance of domain walls is set as *d*_*w*_. As for the area *S* is equal to the sum of *S*_A_, *S*_B_, *S*_1_ and *S*_2_ (*S* = *S*_A_+*S*_B_ + *S*_1_ + *S*_2_), and *S*_A_+*S*_B_=*d*/*tanα*.

From the literature^27^, domain walls may be exhibit conductivity which can affect energy storage. Under the same certain conditions of two-ends-contact, one-end-contact and non-end-contact domain walls in unit volume, the two-ends-contact domain walls are far less likely to store charges, while one-end-contact ones are unsuitable to energy storage for lower breakdown voltage. Therefore, typical non-end-contact domain walls maybe possess more better characteristics of energy storage.

**Fig. 3:**
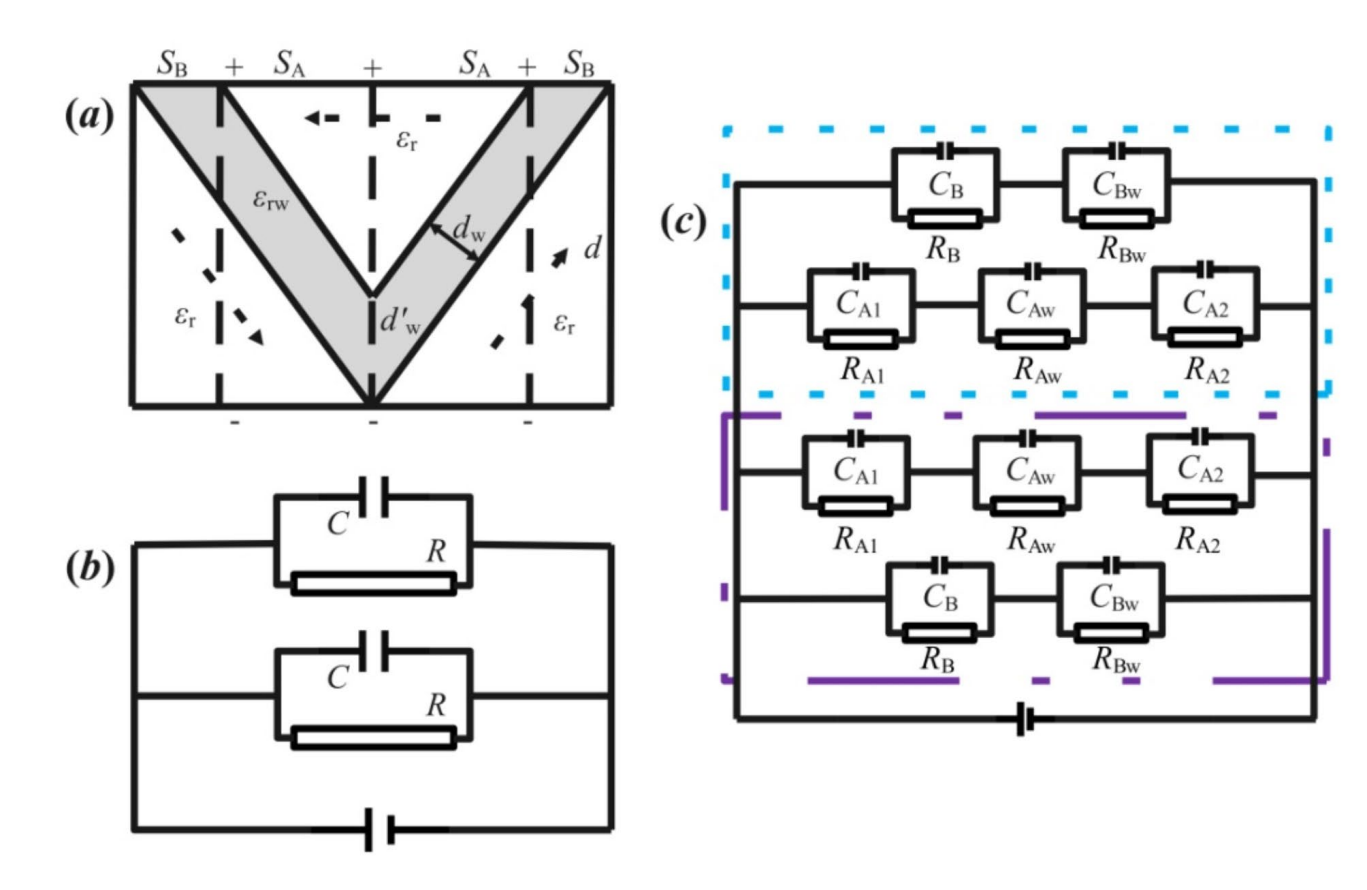
(**a**) is the model diagram of vortex domain structure with bilateral symmetry by three polar domains and two domain walls; (**b**,** c**) are the symmetrical schematic diagram and comprehensive chart of (**a**) equivalent circuit with bilateral symmetry by two different dotted lines, respectively.

As shown in Fig. 3(a) which is corresponding to the Fig. 3(c) in literature^28^ and Fig. 3(d) in literature^29^, three polar domains separated by two domain walls can be divided by dotted lines into the left and right symmetrical parts. Then the equivalent circuit containing two parts of domains and domain walls is illustrated in Fig. 3(b), and the combined capacitance *C*_*T*F3a_= 2*C* is adopted, where *C* can be analyzed from the previous results of *α*-angle structure of domain and domain wall in Fig. 2(a). The specific structure of capacitor *C* is shown in the blue and purple dotted symmetric boxes in Fig. 3(c). The total equivalent capacitance *C*_*T*F3a_ and energy density *w*_*e*F3a_ can be expressed as follows.

10 $$C_{{TF3a}} = 2~C = 2\varepsilon \:_{0} \left[ {\frac{{\varepsilon \:_{r} \varepsilon \:_{{rw}} \left( {\frac{d}{{\tan \alpha \:}} - S_{B} } \right)}}{{\varepsilon \:_{r} d_{w}^{\prime } + \varepsilon \:_{{rw}} \left( {d - d_{w}^{\prime } } \right)}} + \frac{{2\varepsilon \:_{r} \varepsilon \:_{{rw}} S_{B} }}{{\varepsilon \:_{r} d_{w}^{\prime } + \varepsilon \:_{{rw}} \left( {2d - d_{w}^{\prime } } \right)}}} \right]$$

11 $$\:{w}_{e\text{F}3\text{a}}=\frac{{{\epsilon\:}_{0}E}^{2}d}{S}\left[\frac{{\epsilon\:}_{r}{\epsilon\:}_{rw}\left(\frac{d}{tan\alpha\:}-{S}_{B}\right)}{{\epsilon\:}_{r}{d}_{w}^{{\prime\:}}+{\epsilon\:}_{rw}\left(d-{d}_{w}^{{\prime\:}}\right)}+\frac{2{\epsilon\:}_{r}{\epsilon\:}_{rw}{S}_{B}}{{\epsilon\:}_{r}{d}_{w}^{{\prime\:}}+{\epsilon\:}_{rw}\left(2d-{d}_{w}^{{\prime\:}}\right)}\right]$$

**Fig. 4:**
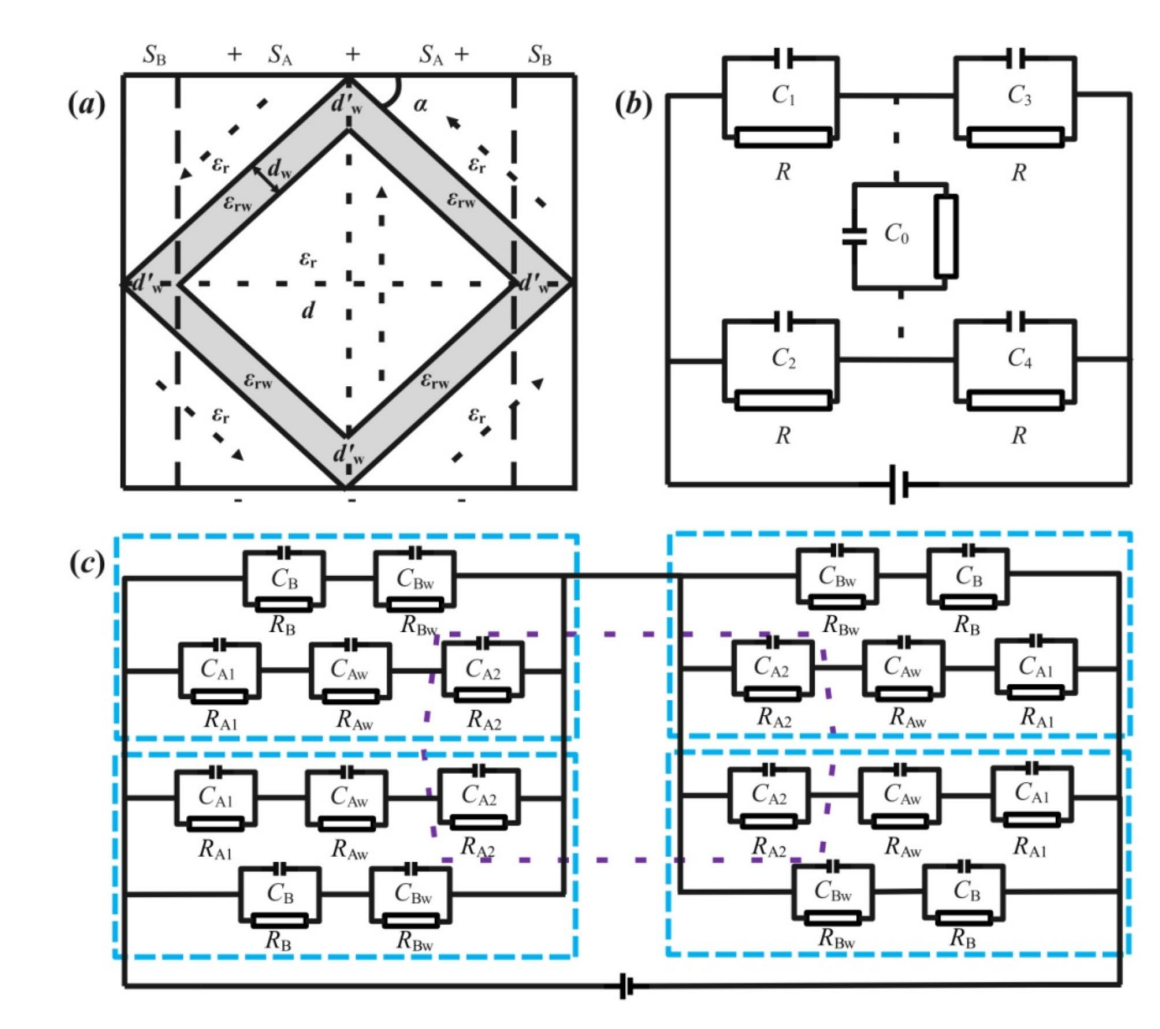
(**a**) is the model diagram of vortex domain structure with quadruple symmetry by five polar domains and four domain walls; (**b**,** c**) are the symmetrical schematic diagram and comprehensive chart of (**a**) equivalent circuit with quadruple symmetry by four dotted lines, respectively, and the fifth capacitor of the central domain in (**b**) can be consisted by the central sub-capacitors enclosed by dotted line in (**c**).

For the assumption of topological domain nanostructures like skyrmion lattices with vortex patterns of the Fig. 1 in literature^28^, the equivalent circuit model of the least five polar domains and four domain walls is illustrated in Fig. 4(a)^28,29^, similar to those with three polar domains in Fig. 3(a). By dotted lines, the whole region of grain is divided into the up, down, left and right four same sub-regions, in which each sub-region contains two *α*-angle domains and one-end-contact type domain wall. Therefore, the equivalent circuit can be shown as Fig. 4(b). Where *d* is the total distance between two pole plates of equivalent capacitor including all domains and domain walls, and *S* is the total opposite area.

Combined with the previous results for one end in contact *α*-angle between domain and domain wall, the total equivalent capacitance *C*_*T*F4a_ and energy density *w*_*e*F4a_ can be deduced from equivalent circuit diagram in Fig. 4(b) and may be illustrated as follow formulas.

12 $$\:{C}_{T\text{F}4\text{a}}=C={\epsilon\:}_{0}\left[\frac{{\epsilon\:}_{r}{\epsilon\:}_{rw}\left(\frac{d}{tan\alpha\:}-{S}_{B}\right)}{{\epsilon\:}_{r}{d}_{w}^{{\prime\:}}+{\epsilon\:}_{rw}\left(d-{d}_{w}^{{\prime\:}}\right)}+\frac{2{\epsilon\:}_{r}{\epsilon\:}_{rw}{S}_{B}}{{\epsilon\:}_{r}{d}_{w}^{{\prime\:}}+{\epsilon\:}_{rw}\left(2d-{d}_{w}^{{\prime\:}}\right)}\right]$$

13 $$w_{{eF4a}} = \frac{{\varepsilon \:_{0} E^{2} d}}{{2~S}}\left[ {\frac{{\varepsilon \:_{r} \varepsilon \:_{{rw}} \left( {\frac{d}{{\tan \alpha \:}} - S_{B} } \right)}}{{\varepsilon \:_{r} d_{w}^{\prime } + \varepsilon \:_{{rw}} \left( {d - d_{w}^{\prime } } \right)}} + \frac{{2\varepsilon \:_{r} \varepsilon \:_{{rw}} S_{B} }}{{\varepsilon \:_{r} d_{w}^{\prime } + \varepsilon \:_{{rw}} \left( {2d - d_{w}^{\prime } } \right)}}} \right]$$

As far as the general case be concerned, when the four regions divided by the polar domains are not proportional, it can be divided into four different sub-regions using the similar method shown in Fig. 4(a). By the fact that the four regions are connected together, its total capacitance can be calculated in consideration of the equivalent Wheatstone-like bridge circuit model, including the dotted section in Fig. 4(b). As for the central equivalent sub-capacitors *C*_0_ in Fig. 4(b), it has been also shown in the purple dotted frame at the center of Fig. 4(c), in which consisting of a portion of four equivalent capacitors *C*_*A*2_. In order to facilitate the distinction, the four capacitors *C* are marked as *C*_1_ ~ C_4_ according to their place sequences in Fig. 4(b): upper left, lower left, upper right and lower right. Referring to the bridge circuit and imagining that *C*_4_ can be divided into two components of *C*_41_ and *C*_42_, *C*_0_and *C*_41_ can be connected in parallel and further connected with *C*_2_ in series^23,30^. Furthermore, the ratio of the sum capacitance of C_2_, C_0_ and C_41_ to that of *C*_42_ is proportional to the ratio of *C*_1_ to *C*_3_. By the equivalent circuit analysis, here the total capacitance *C’*_*T*F4a_ and energy density *w’*_*e*F4a_ are listed as follows.

14 $$\:{C}_{T\text{F}4\text{a}}^{{\prime\:}}=\frac{\left[{\text{C}}_{1}+\frac{{\text{C}}_{2}\left({\text{C}}_{0}+{\text{C}}_{41}\right)}{{\text{C}}_{2}+{\text{C}}_{0}+{\text{C}}_{41}}\right]\left({\text{C}}_{3}+{\text{C}}_{42}\right)}{{\text{C}}_{1}+{\text{C}}_{3}+{\text{C}}_{42}+\frac{{\text{C}}_{2}\left({\text{C}}_{0}+{\text{C}}_{41}\right)}{{\text{C}}_{2}+{\text{C}}_{0}+{\text{C}}_{41}}}$$

15 $$w_{{eF4a}}^{\prime } = \frac{{dE^{2} \left[ {C_{1} + \frac{{C_{2} \left( {C_{0} + C_{{41}} } \right)}}{{C_{2} + C_{0} + C_{{41}} }}} \right]\left( {C_{3} + C_{{42}} } \right)}}{{2~S\left[ {C_{1} + C_{3} + C_{{42}} + \frac{{C_{2} \left( {C_{0} + C_{{41}} } \right)}}{{C_{2} + C_{0} + C_{{41}} }}} \right]}}$$

As like the previous analysis of physical model, the value of capacitor *C*_4_ satisfies as the same value as the sum of equivalent two small *C*_41_ and *C*_42_with certain relations in series. In addition, the ratio of the capacitance *C*_42_ to the total capacitance of *C*_2_, *C*_0_ and *C*_41_ in series, is as the same as that of *C*_3_ to *C*_1_, therefore

16 $$\:\frac{1}{{C}_{4}}=\frac{1}{{C}_{41}}+\frac{1}{{C}_{42}}$$

17 $$\frac{{C_{1} }}{{C_{3} }} = \frac{{C_{2} \left( {C_{0} + C_{{41}} } \right)}}{{C_{2} + C_{0} + C_{{41}} }}/C_{{42}}$$

After simplification, it can be obtained as follows.

18 $$\:{C}_{41}=\frac{\sqrt{{\left[{C}_{1}{C}_{4}\left({C}_{0}+{C}_{2}\right)-{C}_{2}{C}_{3}\left({C}_{0}-{C}_{4}\right)\right]}^{2}-4{C}_{0}{C}_{2}{C}_{3}{C}_{4}\left({C}_{1}{C}_{4}-{C}_{2}{C}_{3}\right)}}{2\left({C}_{1}{C}_{4}-{C}_{2}{C}_{3}\right)}-\frac{\left[{C}_{1}{C}_{4}\left({C}_{0}+{C}_{2}\right)-{C}_{2}{C}_{3}\left({C}_{0}-{C}_{4}\right)\right]}{2\left({C}_{1}{C}_{4}-{C}_{2}{C}_{3}\right)}$$

19 $$\:{C}_{42}=\frac{{C}_{4}{C}_{41}}{{C}_{41}-{C}_{4}}$$

Benefitting from the central resistance in the equal circuits of bridge model, and when the electric potential difference is zero between both ends of central domain in the single-layer plane, the central domain has no charges stored and its capacitance *C*_0_ can be considered equivalent as zero mathematically rather than physically. If the electric potential difference is not zero between both ends of central domain along the perpendicular direction of the single-layer plane, the central domain has some stored charges and its capacitance defined *C*_0_’ is not equivalent as zero. From above three-dimensional analysis, the spatial topological structure can be formed by *C*_0_’ and *C*_0_ and may be utilized for energy storage.

**Fig. 5:**
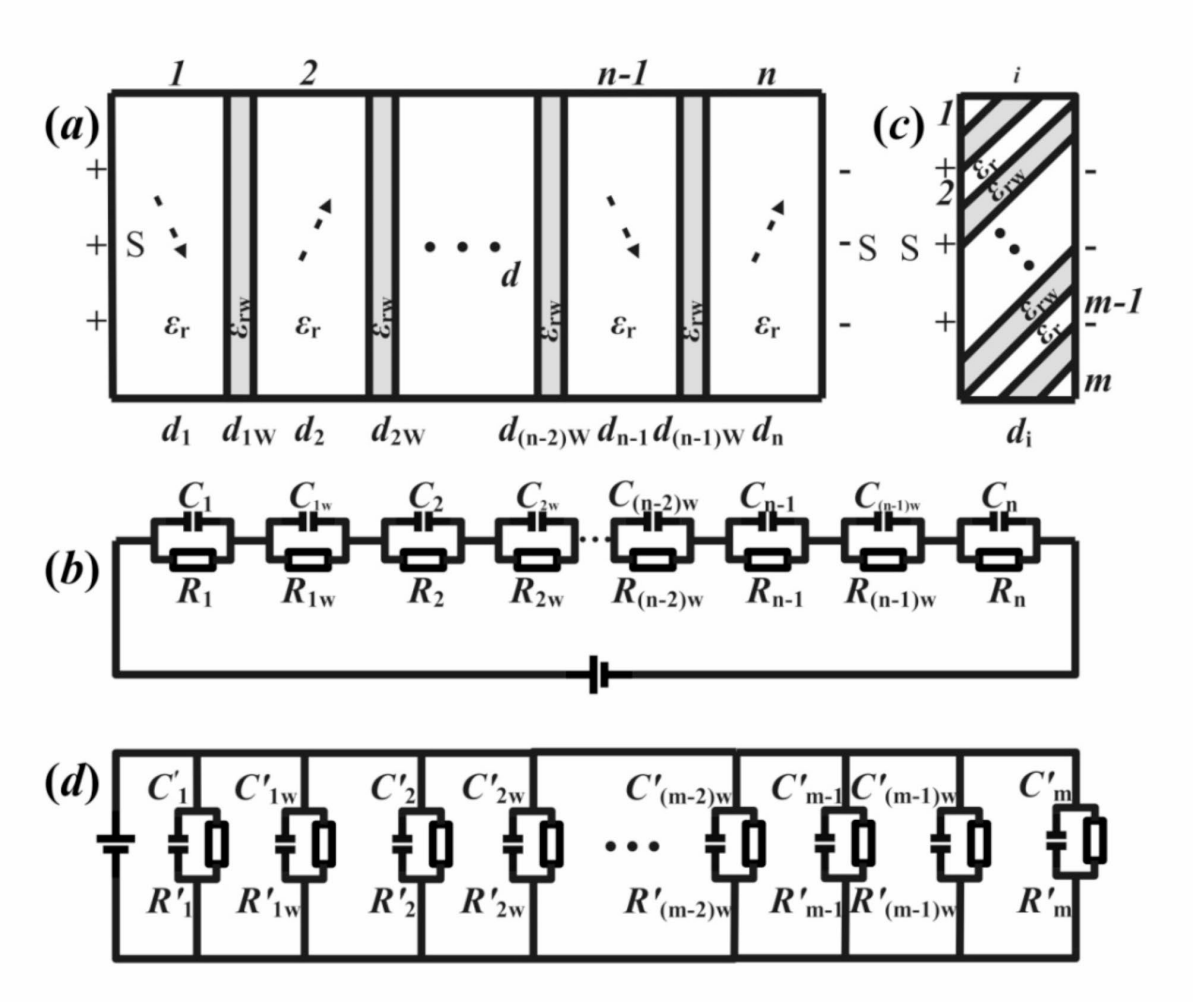
(a, b) are the model diagram and its equivalent circuit of microdomains and domain walls; (c, d) are the model diagram and its equivalent circuit of nanodomains and domain walls in microdomains.

Another way to enhance energy density is by the transformation from macro/microdomain to nanodomain. The experimental performance has been reported in the Fig. 1 of later literature^31^. As shown within Fig. 5(a&c), they have two models for multiple polar domains and the transformations of microdomains to nanodomains. For the condition of multiple domain walls, ***n*** polar domains and (***n*-1**) intermediate domain walls are characterized as blank spaces and gray ones by black lines between them, respectively. And the angles between adjacent domains are given as the same *α*. In Fig. 5(a), the physical model can be regarded as a parallel connection of equivalent capacitors, including those of *α*-angled ***n*** domains and (***n*-1**) domain walls. Correspondingly, its equivalent circuit diagram is designed as Fig. 5(b).

From Fig. 5(b), its total equivalent capacitance *C*_F5a_ and energy density *w*_*e*F5a_ can be deduced as following formulas.

20 $$\:\frac{1}{{C}_{\text{F}5\text{a}}}=\sum\:_{i=1}^{n-1}\left(\frac{1}{{C}_{i}}+\frac{1}{{C}_{iw}}\right)+\frac{1}{{C}_{n}}$$

21 $$w_{{eF5a}} = \frac{{\left[ {\mathop \sum \nolimits_{{i = 1}}^{{n - 1}} \left( {d_{i} + d_{{iw}} } \right) + d_{n} } \right]E^{2} }}{{2S\left[ {\mathop \sum \nolimits_{{i = 1}}^{{n - 1}} \left( {\frac{1}{{C_{i} }} + \frac{1}{{C_{{iw}} }}} \right) + \frac{1}{{C_{n} }}} \right]}}$$

The values of *C*_1_, *C*_2_, … *C*_n_ and *C*_1w_, *C*_2w_, … *C*_(*n*−1)w_ can be taken, considering the detailed experimental conditions of two-end-contact type *α*-angle domain and domain wall model.

As the macrodomains or microdomains marked by blank spaces in Fig. 5(a) can be inset with nano ones as shown in Fig. 5(c).^31^ The equivalent circuit of Fig. 5(c) are regarded as a series of equivalent capacitors for *α*-angle (1, m) nanodomains and (2, m-1) nanodomain walls with one end in contact and the other end not, and of those for *α*-angle (m-2) nanodomains and nanodomain walls with two ends in contact. In further, the corresponding equivalent circuit diagram is shown in Fig. 5(d), and the formulas for sub-total equivalent capacitance *C*_F5c_=*C*_i_ and energy density *w*_*e*F5c_=*w*_*ei*_ are expressed as follows.

22 $$\:{C}_{\text{F}5\text{c}}={C}_{\text{i}}=\sum\:_{j=1}^{m-1}\left({C}_{j}^{{\prime\:}}+{C}_{jw}^{{\prime\:}}\right)+{C}_{m}^{{\prime\:}}$$

23 $$w_{{eF5c}} = w_{{ei}} = \frac{{\left[ {\sum {_{{j = 1}}^{{m - 1}} } \left( {C_{j}^{\prime } + C_{{jw}}^{\prime } } \right) + C_{m}^{\prime } } \right]d_{i} E^{2} }}{{2~S}}$$

There, specific values of *C*^*’*^_1_ to *C*^*’*^_m_ can be given from the detailed measurements or indexed from documents of some samples in the future. Finally, the total equivalent capacitance *C*_F5a_ and energy density *w*_*e*F5a_ in Fig. 5(a) can be instead by following formulas incorporating with those of nanodomains and nanodomain walls from Fig. 5(c), and then are obtained as follows.

24 $$\:\frac{1}{{C}_{\text{F}5\text{a}}}=\sum\:_{i=1}^{n-1}\left[\frac{1}{\sum\:_{j=1}^{m-1}\left({C}_{ij}^{{\prime\:}}+{C}_{ijw}^{{\prime\:}}\right)+{C}_{im}^{{\prime\:}}}+\frac{1}{{C}_{iw}}\right]+\frac{1}{\sum\:_{j=1}^{m-1}\left({C}_{nj}^{{\prime\:}}+{C}_{njw}^{{\prime\:}}\right)+{C}_{nm}^{{\prime\:}}}$$

25 $$w_{{eF5a}} = \frac{{\left[ {\sum {_{{i = 1}}^{{n - 1}} } \left( {d_{i} + d_{{iw}} } \right) + d_{n} } \right]E^{2} }}{{2~S\left[ {\sum {_{{i = 1}}^{{n - 1}} } \left( {\frac{1}{{\sum {_{{j = 1}}^{{m - 1}} } \left( {C_{{ij}}^{\prime } + C_{{ijw}}^{\prime } } \right) + C_{{im}}^{\prime } }} + \frac{1}{{C_{{iw}} }}} \right) + \frac{1}{{\sum {_{{j = 1}}^{{m - 1}} } \left( {C_{{nj}}^{\prime } + C_{{njw}}^{\prime } } \right) + C_{{nm}}^{\prime } }}} \right]}}$$

Generally because of the greater formation energies of domain walls than those of domains^32^, for the transitions of micro-to-nano domains in unit volume, the maximization of number density for parallel non-end contact domain walls is more favorable for energy storage. Hence, the thinner non-end contact domain walls and greater number density in unit volume, the more advantageous it is to improve energy storage density.

## Conclusion

In conclusion, it has been provided valuable insights for the dielectric energy storage behavior, through the utilization of physical transformation from the configurations of ferroelectric domain and domain wall into equivalent circuit models. By categorizing the domain walls into two groups, namely 180° and *α*-angle, and by constructing their equivalent circuit models respectively, the energy storage properties of vortex domains and transformations of micro-to-nano domains have been thoroughly investigated and analyzed. Several important rules have been noted from the results given by formulas. Generally, the energy storage densities of the dielectrics are directly proportional to the total relative permittivity (*ε*_*r*_) of dielectrics within capacitor under the same applied electric field (*E*). Ulteriorly, the energy storage densities of 180° and *α*-angle structures without two-end-contact between domain and domain wall, are inversely proportional to the sum of quotients of their respective distances versus their products of respective relative permittivity (*ε*_*r*_) and the total distance. However, those of *α*-angle structures with two-end-contact are directly proportional to the sum of quotients of their product of respective areas and relative permittivity (*ε*_*r*_) versus the total area. Typical non-end-contact domain walls maybe possess more better characteristics of energy storage. Finally, the energy densities are analyzed as seriously depending on their microstructures like patterns in ferroelectrics. Either for the topological nanostructures with the unique feature of zero polarization in central domain among single plane of vortex pattern, or for the transformations from microdomains to nanodomains within microdomains, increasing their number densities by thinner domain and domain wall can be helpful to enhance their energy storage densities from the derived formulas. The proposed equivalent physical models and analysis methods offer deep comprehension on the energy storage mechanisms, and should be advantageous to further physical research and technical measurement on relative energy science.

## Data Availability

All data generated or analysed during this study are included in this published article.
